# L-Arginine Enhances Oral Keratinocyte Proliferation under High-Glucose Conditions via Upregulation of *CYP1A1*, *SKP2*, and *SRSF5*

**DOI:** 10.3390/molecules28207020

**Published:** 2023-10-10

**Authors:** Junhe Shi, Trevor R. Leonardo, Chen Han, Hiba I. Bangash, Dandan Chen, Harsh M. Trivedi, Lin Chen

**Affiliations:** 1 NMPA Key Laboratory for Clinical Research and Evaluation of Traditional Chinese Medicine, Xiyuan Hospital, China Academy of Chinese Medical Sciences, Beijing 100091, China; junhe_shi@hotmail.com; 2 Department of Periodontics, College of Dentistry, University of Illinois Chicago, Chicago, IL 60612, USA; tleona3@uic.edu (T.R.L.); chan206@uic.edu (C.H.); hibangash3@gmail.com (H.I.B.); 3 Center for Wound Healing and Tissue Regeneration, University of Illinois Chicago, Chicago, IL 60612, USA; 4 Colgate-Palmolive Company, Piscataway, NJ 08854, USA; dandan_chen@colpal.com (D.C.); harsh_m_trivedi@colpal.com (H.M.T.)

**Keywords:** L-Arginine, high glucose, oral keratinocyte, proliferation, *CYP1A1*, *SKP2*, *SRSF5*

## Abstract

High glucose inhibits oral keratinocyte proliferation. Diabetes can lead to delayed oral wound healing and periodontal disease. L-Arginine, one of the most versatile amino acids, plays an important role in wound healing, organ maturation, and development. In this study, L-Arginine was found to enhance oral keratinocyte proliferation under high-glucose conditions. RNA sequencing analysis discovered a significant number of genes differentially upregulated following L-Arginine treatment under high-glucose conditions. Cytochrome P450 family 1 subfamily A member 1 (*CYP1A1*) was the most significantly upregulated gene at 24 and 48 h after L-Arginine treatment. Gene Ontology enrichment analysis found that cell proliferation- and mitosis-related biological processes, such as mitotic nuclear division, mRNA processing, and positive regulation of cell cycle processes, were significantly upregulated. Pathway enrichment analysis found that S-phase kinase-associated protein 2 (*SKP2*) and serine- and arginine-rich splicing factor 5 (*SRSF5*) were the top upregulated genes in cell cycle and spliceosome pathways, respectively. Indirect immunofluorescent cytochemistry confirmed increased protein levels of *CYP1A1*, *SKP2*, and *SRSF5* after L-Arginine treatment. Knockdown of *CYP1A1*, *SKP2*, and *SRSF5* abolished the enhanced proliferative effect of L-Arginine on oral keratinocytes under high-glucose conditions. In conclusion, L-Arginine enhances oral keratinocyte proliferation under high-glucose conditions via upregulation of *CYP1A1*, *SKP2*, and *SRSF5*, suggesting that supplemental L-Arginine in oral care products may be beneficial for oral tissue repair and regeneration.

## 1. Introduction

Diabetes mellitus and its complications affect millions of people worldwide. The National Diabetes Statistics Report issued by the US Centers for Disease Control and Prevention in 2022 showed that, as of 2019, an estimated 37.3 million people of all ages (11.3% of the US population) had diabetes [1]. Moreover, the World Health Organization estimated that there were more than 400 million people in 2014 suffering from diabetes worldwide [2]. Diabetes has a severe impact on the physical and mental health of those affected and is a huge financial burden on societies. It is a major cause of kidney failure, heart attacks, peripheral artery disease, strokes, blindness, hyperglycemic crisis, neuropathy, retinopathy, chronic food ulcers, and limb amputation. In addition, diabetes delays oral wound healing in animal studies. For example, diabetes has been shown to cause delayed gingival wound re-epithelialization, increased neutrophil infiltration, biofilm formation, reduced collagen formation, and myofibroblast differentiation [3]. Diabetes also inhibited collagen deposition, delayed wound healing, and increased alveolar destruction in tooth extraction wounds [4]. In a cheek mucosal wound model, diabetes led to delayed wound closure, prolonged inflammation with persistently high levels of IL-1β and TNF-α, decreased FGF2, decreased angiogenesis, and collagen deposition [5]. Furthermore, diabetes is closely associated with oral diseases such as periodontal disease, tooth loss, and increased oral fungal infection [6,7,8].

Our recent study demonstrates that high-glucose conditions inhibit oral keratinocyte proliferation and migration [9]. Since keratinocytes are the major cell component in the skin and oral epithelia, with keratinocyte proliferation being critical for re-epithelialization during wound closure [10,11], any strategies that are able to improve keratinocyte proliferation would be beneficial for wound healing, especially in diabetic patients.

L-Arginine (Arg) is considered a nonessential amino acid, but it becomes conditionally essential during times of metabolic stress, such as during wound healing [12,13,14,15,16], organ maturation, and development [17,18]. The sources of Arg needed by the human body are either synthesized by organs such as the intestine, kidney, and liver or supplied from food or supplements. Arg is a precursor molecule involved in the synthesis of proteins and production of important biomolecules such as agmatine, citrulline, creatine, glutamate, nitric oxide (NO), polyamines, proline, putrescine, and urea via multiple pathways involving different enzymes, such as arginase type I/II, nitric oxide synthases, arginine decarboxylase, and arginine glycine amidinotransferase [15,19]. Furthermore, Arg regulates many cellular functions, especially proliferation [20]. Undeniably, Arg is one of the most versatile amino acids.

In this study, Arg was found to promote oral keratinocyte proliferation under high-glucose conditions. RNA sequencing demonstrated that Arg treatment induced significant transcriptomic changes in keratinocytes. In particular, cytochrome P450 family 1 subfamily A member 1 (*CYP1A1*), S-phase kinase-associated protein 2 (*SKP2*), and serine- and arginine-rich splicing factor 5 (*SRSF5*) were genes that were significantly upregulated, while several Gene Ontology (GO) terms and pathways, including cell proliferation and mitosis, were enriched. The inhibition of *CYP1A1*, *SKP2*, and *SRSF5* abolished the enhanced proliferation induced by Arg treatment.

## 2. Results

### 2.1. L-Arginine Enhances Oral Keratinocyte Proliferation

**Figure 1:**
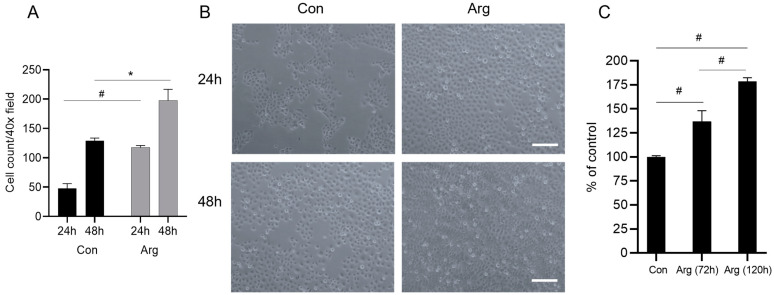
L-Arginine enhances oral keratinocyte proliferation under high-glucose conditions. (**A**) TIGK cells were cultured in 4-chamber slides treated with 500 μM Arg under high-glucose conditions (48 mM). Cell images were taken 24 and 48 h after treatment and the number of cells was counted in each field. (**B**) Representative images of the cultured cells. (**C**) TIGK cells were cultured in 96-well plates. The proliferation was assessed using an MTS proliferation assay. OD490 values were normalized based on the untreated cell condition (100%). Scale bar: 100 µm. Con: Control, Arg: L-Arginine, * *p* < 0.05, # *p* < 0.01.

To assess the effect of Arg in a high-glucose environment on oral keratinocytes, TIGK cells were treated with 500 μM Arg under high-glucose conditions (48 mM). The total number of cells determined by cell count in the Arg-treated group was found to be significantly higher than that in the untreated control (*p* < 0.05–0.01, Figure 1A,B) after 24 and 48 h. Arg treatment was also found to significantly enhance TIGK cell proliferation 72 and 120 h post-treatment as determined by an MTS proliferation assay (*p* < 0.01, Figure 1C). Similar results were obtained using 1 mM Arg. Therefore, 500 μM Arg was used in all of the following experiments. Arg (0.5–2 mM) was also shown to significantly enhance TIGK cell proliferation at low (normal) glucose conditions (6 mM) 72 and 96 h after treatment (Supplementary Materials Figure S1). Due to the clinical relevance to diabetes, the following studies were all performed under high-glucose conditions.

### 2.2. L-Arginine Leads to Significant Transcriptomic Changes in Oral Keratinocytes under High-Glucose Conditions

**Figure 2:**
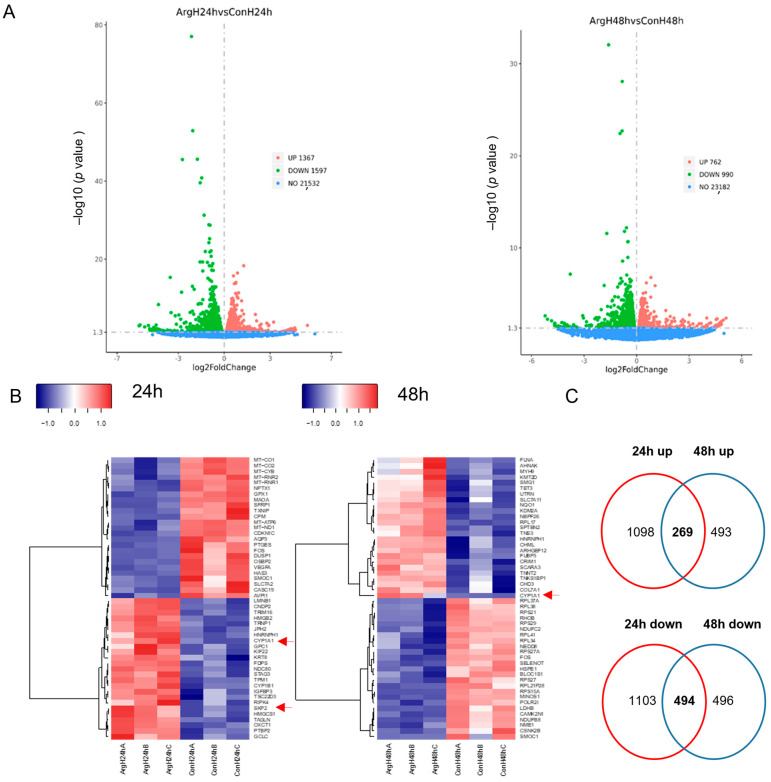
Volcano plots and heat maps derived from RNA sequencing differential expression analyses. (**A**) Volcano plots of the distribution of significantly upregulated, downregulated, and insignificantly differentially expressed genes after Arg treatment (24 h, **left panel** and 48 h, **right panel**). (**B**) Heat maps of the top 25 significantly upregulated and downregulated genes (24 h, **left panel** and 48 h, **right panel**). (**C**) Venn diagrams of overlapping genes between 24 and 48 h upregulated or downregulated genes. Con: Control, Arg: L-Arginine, H: high glucose.

Since Arg was shown to enhance oral keratinocyte proliferation under high-glucose conditions, we further investigated if and to what extent Arg treatment alters the transcriptomics of TIGK cells by performing Poly (A) RNA sequencing at 24 and 48 h after Arg treatment. Differential gene expression analysis, as demonstrated in the volcano plots (Figure 2A), found 1367 and 1597 genes statistically upregulated (red dots) and downregulated (green dots), respectively, 24 h after Arg treatment as compared to the untreated control. There were 762 and 990 genes statistically upregulated and downregulated, respectively, 48 h after treatment as compared to the untreated control. Heat maps of the top 25 differentially expressed genes (DEGs), including upregulated and downregulated genes, at 24 and 48 h after Arg treatment are shown in Figure 2B. The full list of DEGs is listed in Tables S2–S5, respectively. Among the upregulated genes, *CYP1A1* was the most significantly upregulated gene at both 24 and 48 h after treatment (Tables S2 and S4).

When we compared the upregulated or downregulated DEGs between the 24 and 48 h treatment groups, there were 269 and 494 genes found to be commonly differentially expressed at 24 and 48 h, respectively, after Arg treatment (Figure 2C). *CYP1A1*, *SRSF5*, and *SKP2* were among those 269 commonly upregulated genes. The full list of the common upregulated and downregulated DEGs are listed in Tables S6 and S7, respectively.

**Figure 3:**
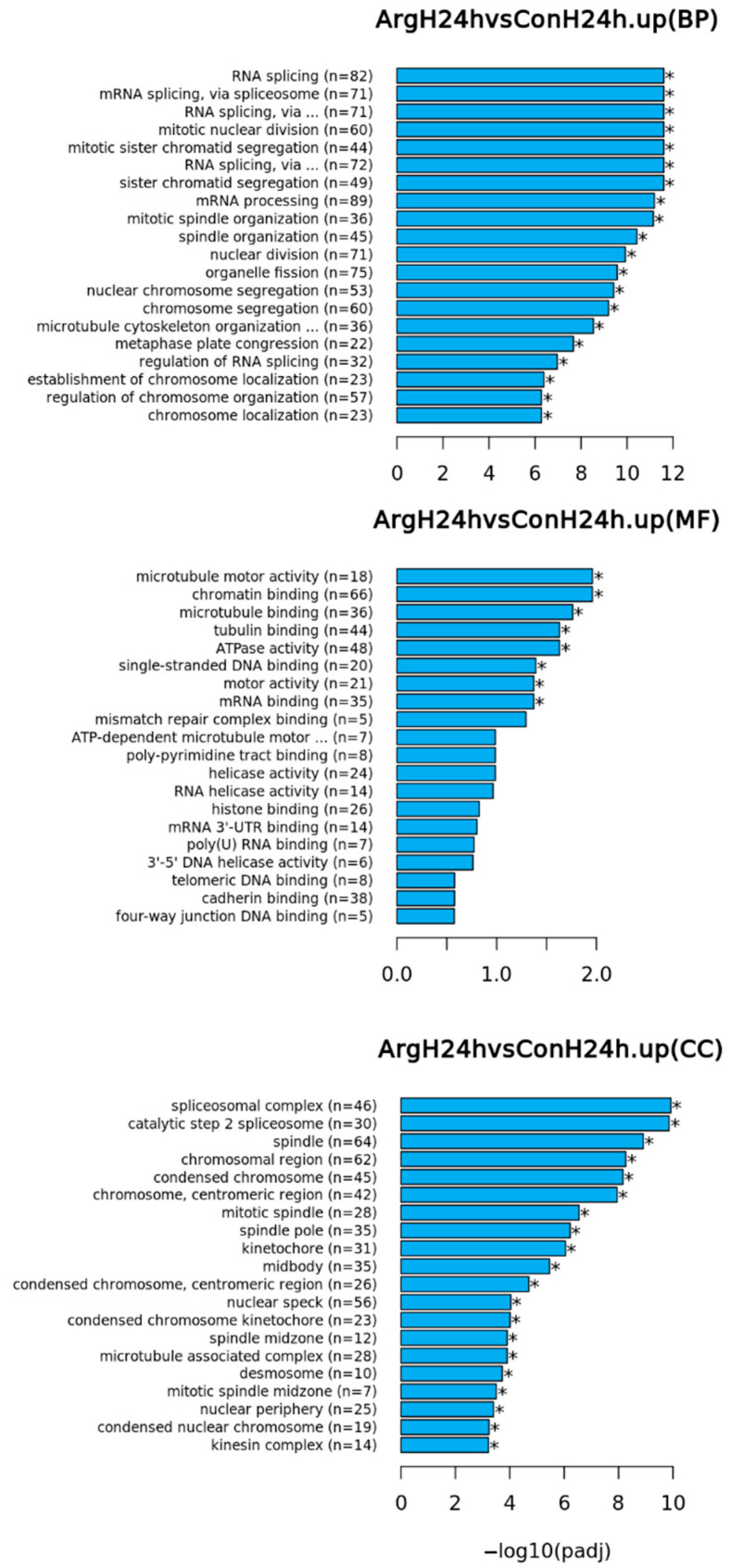
Top 20 gene ontology (GO) terms including biological processes (BP), molecular functions (MF), and cellular components (CC) in differentially upregulated genes 24 h after Arg treatment under high-glucose conditions. Con: Control, Arg: L-Arginine, H: high glucose. * *p* < 0.05.

The results from the differential expression analyses were then subjected to a functional enrichment analysis. There were a number of GO terms significantly changed for BP, MF, and CC at 24 h after Arg treatment under high-glucose conditions. The results are shown in Figure 3 with the top 20 GO terms in each GO category displayed (* *p* < 0.05). For the complete list of significant genes in each term, refer to Table S7. Cell proliferation- and mitosis-related BP terms such as RNA splicing, mitotic nuclear division, sister chromatid segregation, mRNA processing (Figure 3), and positive regulation of cell cycle process (ranked 23rd, Table S8) were significantly upregulated. Similarly, cell proliferation- and mitosis-related MF terms such as microtubule motor activity and chromatin binding and CC terms such as spliceosomal complex, catalytic step 2 spliceosome, and spindle were also significantly upregulated 24 h after treatment (Figure 3 and Table S8). These changes may indicate potential mechanisms which Arg treatment under high-glucose conditions employs to enhance TIGK cell proliferation. There were also a number of GO terms that were significantly downregulated at 24 h (Figure S2 and Table S9) such as cotranslational protein targeting to membrane and oxidative phosphorylation. A number of GO terms were also significantly upregulated or downregulated at 48 h (Figure S3, Tables S10 and S11). However, we do not know if they are relevant to the observed increased TIGK cell proliferation following Arg treatment under high-glucose conditions.

**Figure 4:**
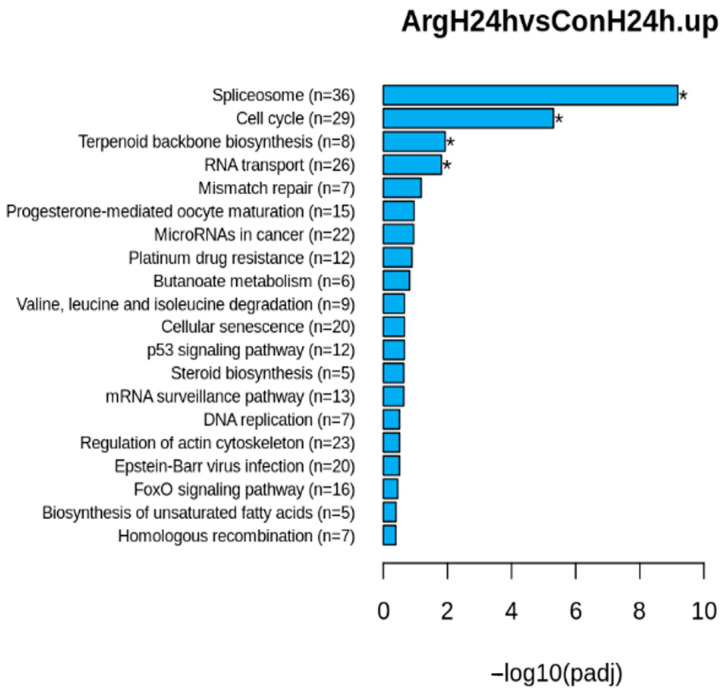
Top 20 Kyoto Encyclopedia of Genes and Genomes (KEGG) pathways enriched in differentially upregulated genes 24 h after L-Arginine treatment under high-glucose conditions. Con: Control, Arg: L-Arginine, H: high glucose. * *p* < 0.05.

The KEGG signaling pathways of cell cycle (Figure S4), spliceosome (Figure S5), and RNA transport (Figure S6) were among the top 20 significant pathways upregulated 24 h after Arg treatment under high-glucose conditions (Figure 4). The gene list of the pathways is listed in Table S12. Spliceosomes remove introns from a transcribed pre-mRNA. RNA transport from the nucleus to the cytoplasm is fundamental for translation to occur. There were 36, 29, and 26 genes that were upregulated in the spliceosome, cell cycle, and RNA transport pathways, respectively (Table S12). Positive regulation of the cell cycle, spliceosome, and RNA transport may explain the observed phenotype of Arg-enhanced cell proliferation. There were no significantly upregulated pathways in cells 48 h after treatment. A number of pathways were also significantly downregulated at either 24 or 48 h after treatment (Figure S7). However, it is not clear if they played any role in the observed increase in TIGK cell proliferation.

*SKP2* is the top gene in the significantly upregulated cell cycle pathway in cells treated with Arg at 24 h (Table S12). It is the 22nd and 47th upregulated DEG at 24 and 48 h, respectively, after treatment (Tables S2 and S4).

*SRSF5* is the top gene in the significantly upregulated spliceosome pathway in cells treated with Arg at 24 h (Table S12). *SRSF5* is the 31st and 422nd upregulated DEG at 24 and 48 h, respectively, after treatment (Tables S2 and S4).

### 2.3. L-Arginine Induces Significant Protein Expression of CYP1A1, SRSF5, and SKP2 under High-Glucose Conditions

**Figure 5:**
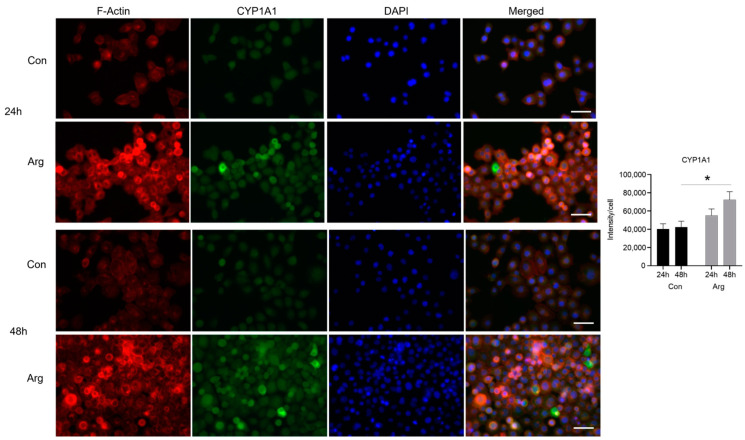
*CYP1A1* in oral keratinocytes is upregulated after L-Arginine treatment under high-glucose conditions. TIGK cells were treated with 500 μM Arg in a culture medium having high glucose (48 mM) for 24 and 48 h. Cells were fixed in formalin and permeabilized with Triton X 100 followed by incubation with a mouse anti–human *CYP1A1* antibody and a goat anti-mouse IgG H&L-Alexa Fluor 488 secondary antibody. Alexa Fluor 594 Phalloidin was used to stain F-actin as a counterstain. DAPI was used for nuclei staining. The green fluorescence intensity of *CYP1A1* was quantified using ImageJ, Version 1.8.0. Scale bar: 50 µm. * *p* < 0.05 compared to control.

**Figure 6:**
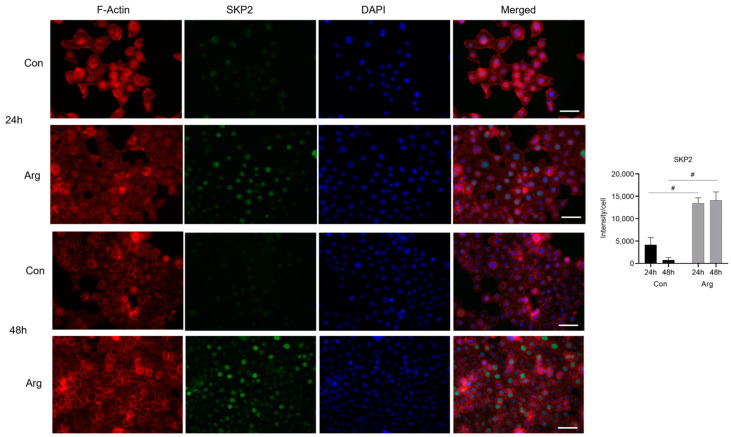
*SKP2* in oral keratinocytes is upregulated after L-Arginine treatment under high-glucose conditions. TIGK cells were treated with 500 μM Arg in a culture medium having high glucose (48 mM) for 24 and 48 h. Cells were fixed in formalin and permeabilized with Triton X-100 followed by incubation with a mouse anti–human *SKP2* antibody and a goat anti-mouse IgG Alexa Fluor 488 secondary antibody. Alexa Fluor 594 Phalloidin was used to stain F-actin as a counterstain. DAPI was used for nuclei staining. The green fluorescence intensity of *SKP2* was quantified using ImageJ. Scale bar: 50 µm. # *p* < 0.01 compared to control.

**Figure 7:**
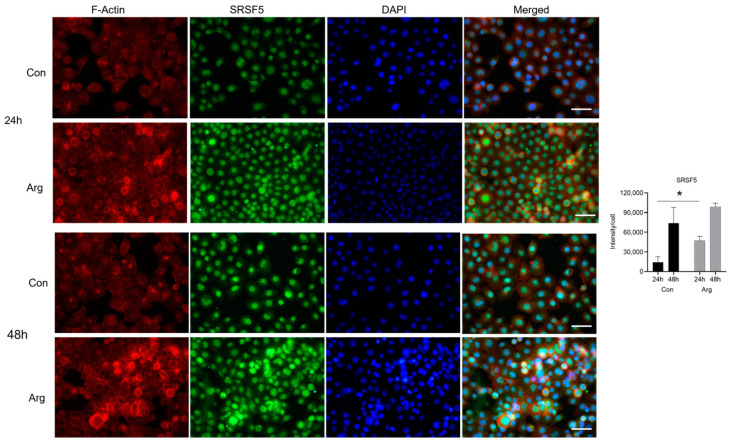
*SKP2* in oral keratinocytes is upregulated after L-Arginine treatment under high-glucose conditions. TIGK cells were treated with 500 μM Arg in a culture medium having high glucose (48 mM) for 24 and 48 h. Cells were fixed in formalin and permeabilized with Triton X-100 followed by incubation with a rabbit anti-human *SKP2* antibody and a goat anti-rabbit IgG Alexa Fluor 488 secondary antibody. Alexa Fluor 594 Phalloidin was used to stain F-actin as a counterstain. DAPI was used for nuclei staining. The green fluorescence intensity of *SKP2* was quantified using ImageJ. Scale bar: 50 µm. * *p* < 0.05 compared to control.

*CYP1A1* was the top upregulated DEG 24 and 48 h after Arg treatment under high-glucose conditions. *SRSF5* and *SKP2* were the top genes in the significantly upregulated cell cycle and spliceosome pathways 24 h after Arg treatment, respectively. We used immunofluorescence to examine if the protein levels of these molecules were elevated after treatment. As shown in Figure 5, the fluorescence intensity of cytoplasmic staining of *CYP1A1* in TIGK cells was significantly increased after Arg treatment compared to the untreated control under high-glucose conditions, especially at 48 h (Figure 5, *p* < 0.05). The fluorescence intensity of *SKP2* in the nuclei of TIGK cells was significantly increased at 24 and 48 h after Arg treatment compared to the untreated control under high-glucose conditions (Figure 6, *p* < 0.01 at 24 and 48 h). Similarly, the expression of *SKP2* in the nuclei of TIGK cells after Arg treatment was also markedly increased compared to the untreated control, especially at 24 h (Figure 7, *p* < 0.05). These results validate the increased gene expression observed in the RNA sequencing analysis described above.

### 2.4. Knockdown of CYP1A1, SRSF5, and SKP2 Abolishes Enhanced Proliferation of Oral Keratinocytes by L-Arginine under High-Glucose Conditions

**Figure 8:**
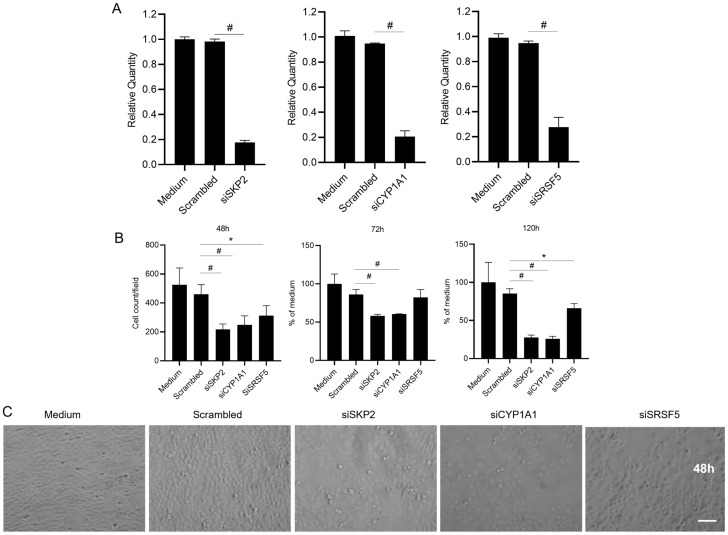
Knocking down *CYP1A1*, *SKP2*, or *SRSF5* abolishes enhanced oral keratinocyte proliferation induced by L-Arginine treatment under high-glucose conditions. TIGK cells were transfected with 20 nM siRNAs of *CYP1A1*, *SKP2*, or *SRSF5* and then treated with 500 μM Arg in a culture medium with high glucose (48 mM). (**A**) mRNA expression of *CYP1A1*, *SKP2*, or *SRSF5* 48 h after transfection. (**B**) The proliferation of TIGK cells after knocking down *CYP1A1*, *SKP2*, or *SRSF5* assessed by cell count at 48 h and MTS assay at 72 and 120 h after transfection. (**C**) Representative images of cell density at 48 h after treatment. Scale bar: 100 µm. * *p* < 0.05, # *p* < 0.01.

*CYP1A1*, *SRSF5*, and *SKP2* were identified as either top DEGs or top genes involved in the cell cycle or spliceosome pathways. The data described above show that both gene and protein levels of these molecules were significantly elevated by treatment with Arg under high-glucose conditions. To assess the degree to which these genes affect these processes, TIGK cells were transfected with siRNAs targeting *CYP1A1*, *SRSF5*, or *SKP2*. The siRNA transfections successfully significantly inhibited mRNA expression of *CYP1A1*, *SRSF5*, and *SKP2* compared to a scrambled siRNA control 48 h after transfection (*p* < 0.01, Figure 8A). There was no significance between the medium-only control and scrambled siRNA (*p* > 0.05, Figure 8A). Knockdown of any three of these targets was also shown to significantly impede the proliferation of TIGK cells at 48 and 120 h after transfection compared to the scrambled siRNA control (*p* < 0.05–0.01, Figure 8B,C). Knockdown of *SKP2* and *CYP1A1*, but not *SRSF5*, also significantly inhibited the proliferation of TIGK cells at 72 h after transfection (*p* < 0.01, Figure 8B). The results confirm that these three molecules play critical roles in the enhanced proliferation induced by Arg treatment under high-glucose conditions.

## 3. Materials and Methods

### 3.1. Cell Culture

Human telomerase immortalized gingival keratinocytes (TIGK) (ATCC, Catalog # CRL-3397) were propagated in DermaLife K Keratinocyte Complete Medium (Lifeline Cell Technology, Frederick, MD, USA, Catalog # LL-0007) containing D-Glucose (6 mM), insulin (5 µg/mL), L-Glutamine (6 mM), epinephrine (1 µM), apo-transferrin (5 µg/mL), TGF-α (0.5 ng/mL), pituitary extract (0.4%), and hydrocortisone hemisuccinate (100 ng/mL). DMEM-SILAC Flex (ThermoFisher Scientific, Waltham, MA, USA, Catalog # A2493901), which contained no Arg, L-Glutamine, L-lysine, or D-Glucose, was used as a base medium for the following assays. Supplemental nutrients or growth factors including insulin (5 µg/mL), L-Glutamine (6 mM), epinephrine (1 µM), apo-transferrin (5 µg/mL), TGF-α (0.5 ng/mL), pituitary extract (0.4%), hydrocortisone hemisuccinate (100 ng/mL) (Lifeline Cell Technology), L-lysine (0.798 mM, Sigma-Aldrich, St. Louis, MO, USA, Catalog # 44208)), and D-Glucose (6 mM or 48 mM, Sigma-Aldrich, Catalog # G7021) were added to the medium as needed. TIGK cells were cultured at 37 °C with 5% CO_2_.

For the proliferation assay, when TIGK cells reached 80% confluency in a flask, TrypLE (Thermo Fisher Scientific) was used to detach the cells. A total of 5000 cells/well for the 24, 48, 72, and 96 h treatments and 3000 cells/well for the 120 h treatment were seeded in 96-well plates. After 12 h, cells were treated with Arg (500 and 1000 μM, Sigma-Aldrich, Catalog # A5006) in 200 μL medium under low-glucose (6 mM) or high-glucose conditions (48 mM). For the 120 h test, the culture medium was changed once at 72 h post-treatment. Cell proliferation was examined using an MTS Cell Proliferation Assay Kit (Abcam, Waltham, MA USA, Catalog # ab197010), according to the manufacturer’s instructions, at 24, 72, and 96 h post-treatment for low-glucose (6 mM) conditions or 72 and 120 h post-treatment for high-glucose (48 mM) conditions. OD_490_ values were recorded using a spectrophotometer (Molecular Devices, San Jose, CA, USA). The number of cells per field for 24 and 48 h post-treatment for high-glucose (48 mM) conditions was manually counted. All treatment groups had three to four replicates. The results are shown as the percent OD_490_ value relative to the control OD_490_ values at each time point.

### 3.2. RNA Sequencing and Bioinformatics Analysis

Arg treatment: When TIGK cells reached 80% confluency in a culture flask, TrypLE was used to detach the cells. A total of 3 × 10^5^ cells were seeded in each well of a 6-well plate. After 24 h, cells were treated with Arg (500 μM) for 24 or 48 h under high-glucose (48 mM) conditions. Each treatment condition had three replicates (A, B, and C, Table S1).

RNA isolation, library construction, sequencing, and data analysis: Cells were dissociated from 6-well plates using TrypLE, washed twice with cold 1× PBS, and centrifuged at 175× *g* for 5 min at 4 °C. Total RNA was extracted, and genomic DNA was removed using an RNeasy Plus Mini kit (Qiagen, Hilden, Germany, Catalog # 74034). RNA concentrations and RNA integrity were assessed using an RNA Nano 6000 Assay Kit on an Agilent Bioanalyzer 2100 system (Agilent Technologies, Wood Dale, IL, USA). A summary of the RNA concentrations and RNA integrity numbers (RIN) is shown in Table S1. RNA (1 µg per sample) was used as input for sequencing library generation using the NEBNext Ultra Library Prep Kit for Illumina (Omaha, NE, USA). The quality of the prepared libraries was assessed using an Agilent Bioanalyzer 2100 DNA Assay Kit (Agilent Technologies) before being run on an Illumina sequencing platform (Illumina). For each sample, approximately 6 GB of raw data of 150 bp paired-end reads were generated. Quality control was performed using fastp [21] to remove adapter-contaminated or poly-N sequences and reads of low quality. Cleaned, high-quality reads were mapped to the Homo Sapiens reference genome (GRCh38/hg38) using Spliced Transcripts Alignment to a Reference (STAR) [22] software, annotated using FeatureCounts [22], and Fragments Per Kilobase of exon model per Million mapped reads (FPKM) [23] was calculated. Differential expression analysis for each two-group comparison was performed using the DESeq2 R package [24], and resulting *p*-values were adjusted using the Benjamini and Hochberg’s approach [25] to control for False Discovery Rate (FDR). For each comparison, genes with a *p*-value < 0.05 were determined to be differentially expressed. Gene Ontology (GO) enrichment analysis and Kyoto Encyclopedia of Genes and Genomes (KEGG) pathway enrichment analysis of differentially expressed genes were independently performed using the clusterProfiler R [26] package. GO and KEGG terms with corrected *p*-values < 0.05 were considered statistically significantly enriched terms. Functional enrichment analysis allows us to identify which biological functions or pathways are significantly associated with the respective differentially expressed genes. Gene Ontology (GO) annotates genes to biological processes (BP), molecular functions (MF), and cellular components (CC). KEGG annotates genes to pathways. Heatmaps were generated using heatmap3 (v1.1.1) [27]. The RNA sequencing data were deposited in the GEO database with an accession number of GSE223773. RNA sequencing and bioinformatics analysis were performed by Novogene America (Sacramento, CA, USA).

### 3.3. Indirect Immunofluorescent Cytochemistry

Indirect immunofluorescent cytochemistry was used to visualize and quantify the protein expression of *CYP1A1*, *SKP2*, and *SKP2* in TIGK cells after Arg treatment under high-glucose (48 mM) conditions. When TIGK cells reached 80% confluency in a flask, 4 × 10^4^ (for 48 h treatment group) and 6 × 10^4^ (for 24 h treatment group) TIGK cells were seeded in each well of a 4-well chamber slide (Thermo Fisher Scientific, Catalog No: PEZGS0416). After 12 h, cells were treated with 500 µM Arg under a high-glucose condition (48 mM) for either 24 or 48 h, after which cells were then fixed in formalin for 10 min. After two washes with PBS, cells were permeabilized with 0.15% Triton X 100 for 10 min and blocked with 5% goat serum in PBS for 1 h at room temperature and subsequently incubated with mouse anti–human *CYP1A1* (1A3-03) (Santa Cruz Biotechnology INC, Dallas, TX, USA, Catalog No: sc-101828, 1/100 dilution), mouse anti-human *SKP2* (A-2) (Santa Cruz Biotechnology INC, Catalog No: sc-74477, 1/100 dilution), or rabbit anti-human *SKP2* (Proteintech, Rosemont, IL, USA, Catalog No: 16237-1-AP, 1/250) overnight at 4 °C. The negative control was incubated with the same concentrations of mouse IgG (Thermo Fisher Scientific, Catalog # 31903) or rabbit IgG (Pierce Biotechnology, Rockford, IL, USA, Catalog # 31207). After three washes with PBS, cells were treated with either goat anti-rabbit IgG (H+L) cross-adsorbed secondary antibody-Alexa Fluor 488 (Thermo Fisher Scientific, Catalog No: A11008, 1/1000 dilution) or goat anti-mouse IgG H&L-Alexa Fluor 488 (Abcam, Catalog No: ab150113, 1/500 dilution) for 1 h. The cells were simultaneously treated with Alexa Fluor 594 Phalloidin (Thermo Fisher Scientific, Catalog number: A12381) to stain F-actin. After three washes, slides were mounted with a mounting medium containing 50% glycerol in PBS with 4′,6-diamidino-2-phenylindole (DAPI) for nuclei staining. Fluorescent images were acquired using a digital camera (Axiocam MRc, Zeiss, Oberkochen, Germany) connected to an Axioskop 40 microscope with a 40x objective lens (Zeiss). From three to seven images chosen at random were taken for each treatment condition. ImageJ software (National Institutes of Health, Bethesda, MD, USA) was used to quantify the fluorescence intensity. Four black-and-white random images of the cells were also taken using an Axiovert 40 CFL inverted microscope and an Axiocam Erc digital camera (Zeiss) under a 20× lens. These images were used to count cell numbers for assessing cell proliferation at 24 and 48 h after Arg treatment.

### 3.4. siRNA Knockdown of CYP1A1, SKP2, and SRSF5

TIGK cells were propagated in DermaLife K Keratinocyte Complete Medium (Lifeline Cell Technology) without antibiotics. Totals of 1 × 10^5^, 5 × 10^4^, and 5 × 10^3^ cells were seeded in each well of 12-well, 24-well, and 96-well plates, respectively. When cells reached 60% confluency (for 12-well and 24-well plates), cells were washed with an siRNA transfection medium (Santa Cruz Biotechnology, Catalog number: sc-36868) and then incubated with 20 nM siRNAs for *CYP1A1*, *SKP2*, and *SRSF5* (Santa Cruz Biotechnology, Catalog number: sc-41483, sc-36499, and sc-38342, respectively) or with a scrambled siRNA control (Santa Cruz Biotechnology, Catalog number: sc-37007). After 6 h, DermaLife K Keratinocyte Complete Medium with double concentrations of antibiotics and supplements was added. After 24 h, the medium was then switched to DMEM-SILAC Flex (ThermoFisher Scientific) supplemented with the same supplements as in the DermaLife K Keratinocyte Complete Medium as well as with 48 mM D-glucose and 500 μM Arg. Cells from 12-well plates were harvested using TriZol (ThermoFisher Scientific, Catalog number: 15596026) for total RNA extraction. Cells in the 24-well plates were imaged and the number of cells under each field was counted to assess cell proliferation 48 h after transfection. An MTS assay was also performed in 96-well plates 72 and 120 h after transfection.

### 3.5. Real-Time PCR

Total RNA was extracted from siRNA-treated TIGK cells and incubated with DNAse (Qiagen, Catalog number: 79254) to remove the potential contamination of genomic DNA. Total RNA (1 µg from each sample) was converted to cDNA using a High-Capacity Reverse Transcription Kit (Applied Biosystems, Waltham, MA, USA, Catalog number: 4368814) according to the manufacturer’s directions. The mRNA expression of *CYP1A1*, *SKP2*, and *SRSF5* was determined using real-time PCR using specific primers and Power SYBR Green PCR Master Mix (Roche, Basel, Switzerland, Catalog number: 4913850001). A StepOnePlus RealTime PCR System (Applied Biosystems) was used to perform the PCR. Relative expression was calculated using the 2^−∆∆CT^ method using glyceraldehyde 3-phosphate dehydrogenase (*GAPDH*) as a house-keeping gene. A medium control was used as the baseline for comparison. The specific primers for *CYP1A1* were: forward 5′-CACCTCCAAGATCCCTACACTGA-3′, reverse 5′-ACCAGACAGAAGATGACAGAGGC-3′. The primers for *SKP2* were: forward 5′-GGTGTTTGTAAGAGGTGGTA-3′, reverse 5′-GAGACAGTATGCCGTGGA-3′. The primers for *SRSF5* were: forward 5′-AGTGGCTGTCGGGTATTCATC-3′, reverse 5′-CCGTCCATATCCCTTGAAGAATC-3′. The primers for *GAPDH* were: forward 5′-CAGGGCTGCTTTTAACTCTGG-3′, reverse 5′-TGGGTGGAATCATATTGGAACA-3′.

### 3.6. Statistical Analysis

Results are expressed as means ± standard deviations. Statistical analyses (*t*-test) were performed using GraphPad Prism 9.0 software (GraphPad Software). *p*-Values < 0.05 were considered statistically significant.

## 4. Discussion

Arg plays an important role in the proliferation of different cell types, such as lymphocytes [28], intestinal epithelial cells [29], endometrial epithelial cells [30], and fibroblasts [31]. The underlying mechanism is partly due to Arg metabolites such as polyamines and NO which are critical factors for cell proliferation [28,30,32,33,34]. Previous work has shown that a high-glucose environment impairs the migration and proliferation of cultured skin keratinocytes [35,36,37,38]. Our recent study has also shown that high glucose inhibits oral keratinocyte proliferation and migration, induces late keratinocyte differentiation, and causes cell death [9]. Given the pivotal roles of Arg in cell proliferation, we investigated if and how Arg regulated the proliferation of oral keratinocytes especially under high-glucose conditions.

Our study here, for the first time, discovered that Arg enhances the proliferation of oral keratinocytes under both low- and high-glucose conditions. Due to the detrimental pathological effects of high glucose on oral keratinocytes [9] and its clinical relevance to diabetes, our studies were performed under high-glucose conditions. RNA sequencing analyses identified *CYP1A1* as the top upregulated DEG at both 24 and 48 h after Arg treatment. *CYP1A1* is a responsive gene of aryl hydrocarbon receptor (AhR), a ligand-activated transcription factor. Downregulation of AhR or *CYP1A1* prevents the cell cycle transition from G0/G1 to the S phase, which results in inhibition of the proliferation of human skin keratinocytes [39]. Pathway analysis showed that *SKP2* was the top gene in the significantly upregulated cell cycle pathway. *SKP2* is a nuclear protein and a member of the F-box family of ubiquitin–protein ligase complexes. Cellular processes such as cell cycle, cell proliferation, apoptosis, differentiation, and survival are closely related to *SKP2* [40,41]. It promotes cell cycle entry into the S phase and regulates the stability of other cell cycle-related proteins. *SKP2* is also considered an oncogene since *SKP2* is found to be overexpressed in various cancer cells [40,41]. Pathway analysis also showed that *SRSF5* was the top gene in the significantly upregulated spliceosome pathway. *SRSF5*, also known as SRp40 or HRS, is a member of the serine/arginine-rich protein (SR) family. *SRSF5* is a nuclear protein and a part of the ribonucleoprotein complex of the spliceosome, which removes introns from a transcribed pre-mRNA and plays a critical role in the regulation of pre-mRNA splicing [42]. *SRSF5* was reported to promote cell growth, especially in cancer cells, and is, therefore, also considered an oncogene [43,44,45,46]. Another member of the SR family, SRSF6, was found to promote skin hyperplasia [47]. We speculated that these three upregulated genes identified in the RNA sequencing analysis were in part responsible for the observed enhanced TIGK cell proliferation following Arg treatment under high-glucose conditions. Therefore, we used siRNAs specific to *CYP1A1*, *SKP2*, or *SRSF5* to interfere with their expression and function. Our results show that siRNA targeting of *CYP1A1*, *SKP2*, and *SRSF5* significantly inhibits Arg-induced enhanced TIGK cell proliferation, which confirms that *CYP1A1*, *SKP2*, and *SKP2* play pivotal roles in oral keratinocyte proliferation under high-glucose conditions. The mechanism by which Arg upregulates the expression of *CYP1A1*, *SKP2*, and *SRSF5* under high-glucose conditions remains unknown and warrants further studies.

Arg supplementation has been shown to increase collagen deposition, wound breaking strength, and T lymphocyte function in acute human and rat skin wounds [19,48]. Arg-deficient animals had impaired wound healing, and decreased collagen deposition and wound breaking strength [14]. The data suggest that Arg improves wound healing and immune responses in animals and human patients under normal glucose conditions. Patients with oral or laryngeal cancer receiving supplements with Arg had significantly reduced fistula formation and length of post-operative stay [49,50]. Furthermore, Arg together with zinc oxide and zinc citrate protected the barrier integrity of gingival keratinocytes from TNF-α-induced damage and promoted keratinocyte proliferation and migration, which suggests that the combination offers benefits for oral health [51].

Arg is a unique substrate for NO synthesis [12,15]. NO is a critical factor that regulates vascular homeostasis, inflammation, and antimicrobial activity during the wound healing process [52] and has been shown to promote keratinocyte proliferation [32,34]. However, NO production is decreased in diabetic wounds, which partially contributes to the observed impaired wound healing in those affected with diabetes [53]. Supplemental Arg was found to restore previously decreased NO levels in diabetic wound fluid and wound breaking strength [16,54]. In an acute diabetic rat skin wound model, systemic application (gavaged) of Arg resulted in better wound healing and a higher expression of NO in wound fluid than topical application of Arg [55]. Furthermore, clinical studies have found that the use of Arg is beneficial for diabetic skin wound healing. One study showed that the majority of patients with chronic diabetic foot ulcers who received a subcutaneous injection of Arg subsequently had those ulcers healed [56]. Another study showed that chronic diabetic foot ulcers in patients with hypoalbuminemia that received Arg supplementation healed better than non-Arg-supplemented controls [57]. These results suggest that Arg supplementation is beneficial for diabetic wound healing. Our current study shows that Arg treatment enhances oral keratinocyte proliferation under high-glucose conditions with *CYP1A1*, *SKP2*, and *SRSF5* as significantly upregulated genes. Downregulation of any of those three genes ameliorated the pro-proliferation effects of Arg. It is currently unknown whether Arg treatment and the differential expression of *CYP1A1*, *SKP2*, and *SRSF5* lead to the production of NO or other Arg metabolites. Whether this is responsible for the observed enhanced oral keratinocyte proliferation in this study requires further investigation. Based on the results of the current study, using Arg in oral care products may be a good strategy to improve oral health and prevent or promote oral wound healing in diabetic patients. Future clinical studies are warranted to prove this hypothesis. Studies using in vivo animal models of diabetic wound healing will also be needed to confirm the potential beneficial effects observed in our current in vitro study. In addition, future studies will also investigate some of the downregulated GO terms or genes after Arg treatment under high-glucose conditions.

Taken together, Arg significantly enhances oral keratinocyte proliferation under high-glucose conditions. The treatment leads to changes in a substantial number of gene transcripts related to cell cycle, cell proliferation, and spliceosome GO terms as well as signaling pathways. Among these top upregulated genes, *CYP1A1*, *SKP2*, and *SRSF5* are of particular significance as they play important roles in the Arg-stimulated proliferation of oral keratinocytes. The results suggest that supplemental Arg in oral care products may be beneficial for oral tissue repair and regeneration especially under diabetic conditions.

## Data Availability

Data are available upon request to the corresponding author.

## Supplementary Materials

The following supporting information can be downloaded at: https://www.mdpi.com/article/10.3390/molecules28207020/s1, Figure S1: L-Arginine enhances oral keratinocyte proliferation under a low-glucose condition (6 mM); Figure S2: Top 20 downregulated enriched gene ontology (GO) terms (biological processes/BP, molecular functions/MF, and cellular components/CC) 24 h after Arg treatment under high-glucose conditions; Figure S3: Top 20 upregulated and downregulated enriched gene ontology (GO) terms (biological processes/BP, molecular functions/MF, and cellular components/CC) 48 h after Arg treatment under high-glucose conditions; Figure S4: Significantly upregulated cell cycle pathway in enriched Kyoto Encyclopedia of Genes and Genomes (KEGG) 24 h after Arg treatment under high-glucose conditions; Figure S5: Significantly upregulated spliceosome pathway in enriched Kyoto Encyclopedia of Genes and Genomes (KEGG) 24 h after Arg treatment under high-glucose conditions; Figure S6: Significantly upregulated RNA transport in enriched Kyoto Encyclopedia of Genes and Genomes (KEGG) pathway 24 h after Arg treatment under high-glucose conditions; Figure S7: Top 20 downregulated enriched Kyoto Encyclopedia of Genes and Genomes (KEGG) pathways 24 and 48 h after Arg treatment under high-glucose conditions. Table S1: RNA integrity numbers; Table S2: Upregulated differentially expressed genes (ArgH24h vs. ConH24h; Table S3: Downregulated differentially expressed genes (ArgH24h vs. ConH24h); Table S4: Upregulated differentially expressed genes (ArgH48h vs. ConH48h); Table S5: Downregulated differentially expressed genes (ArgH48h vs. ConH48h); Table S6: Venn UP; Table S7: Venn DOWN; Table S8: GO terms 24 h UP; Table S9: GO terms 24 h DOWN; Table S10: GO terms 48 h UP; Table S11: GO terms 48 h DOWN; Table S12: ArgH24h vs.ConH24h.up.KEGGenrich.
