# Supplementary material for: L-Arginine Enhances Oral Keratinocyte Proliferation under High-Glucose Conditions via Upregulation of CYP1A1, SKP2, and SRSF5
Source: Molecules. 2023 Oct 10;28(20):7020. doi: 10.3390/molecules28207020 (PMC10609441; doi:10.3390/molecules28207020)
Supplement: Supplementary file 1 [file molecules-28-07020-s001.zip › Suppl/Suppl figures.pdf]

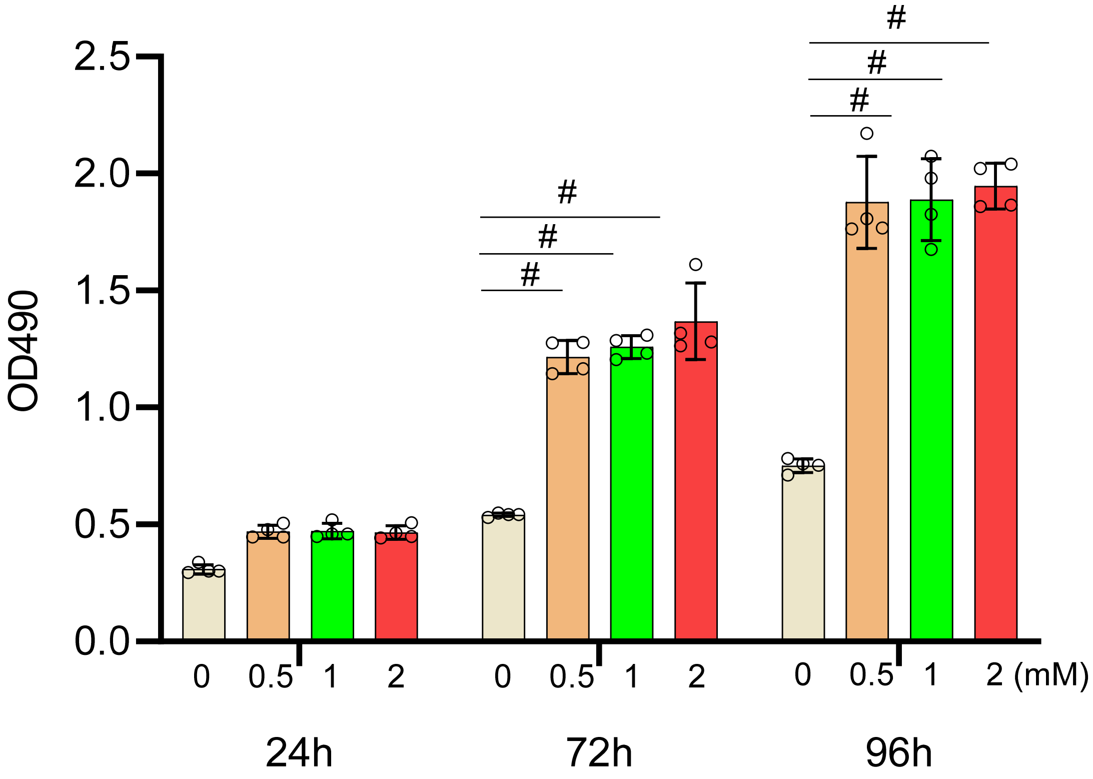

Figure S1: L-Arginine enhances oral keratinocyte proliferation under a low glucose condition (6mM). TIGK cells were treated with Arg with concentrations ranging from 0.5 to 2mM. Proliferation was assessed by an MTS proliferation assay 24-96 hours after treatment. OD490 values were recorded. # $p<0.01$ compared to untreated cells.

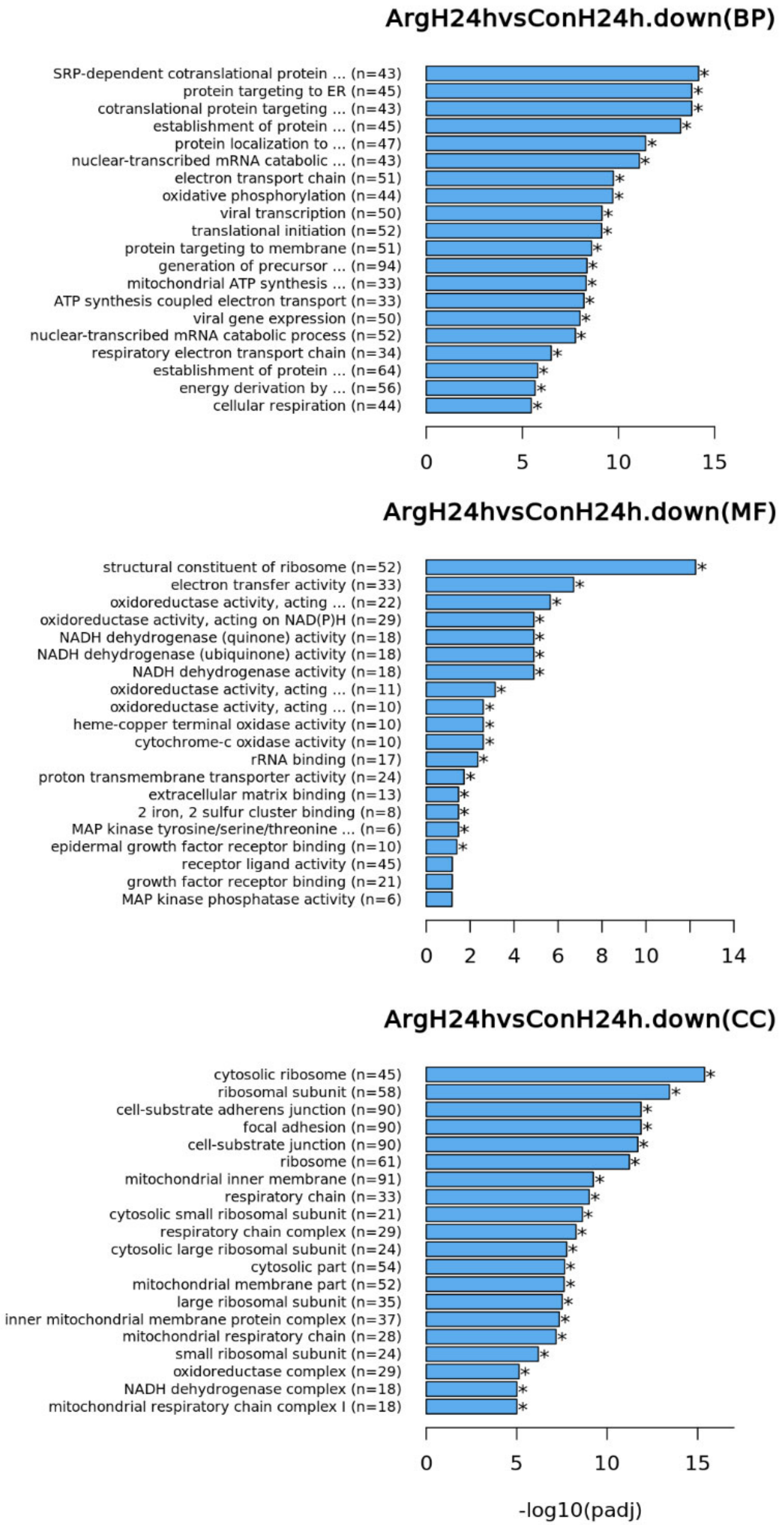

Figure S2: Top 20 downregulated enriched gene ontology (GO) terms (biological processes/BP, molecular functions/MF, and cellular components/CC) 24 hours after Arg treatment under high glucose conditions.

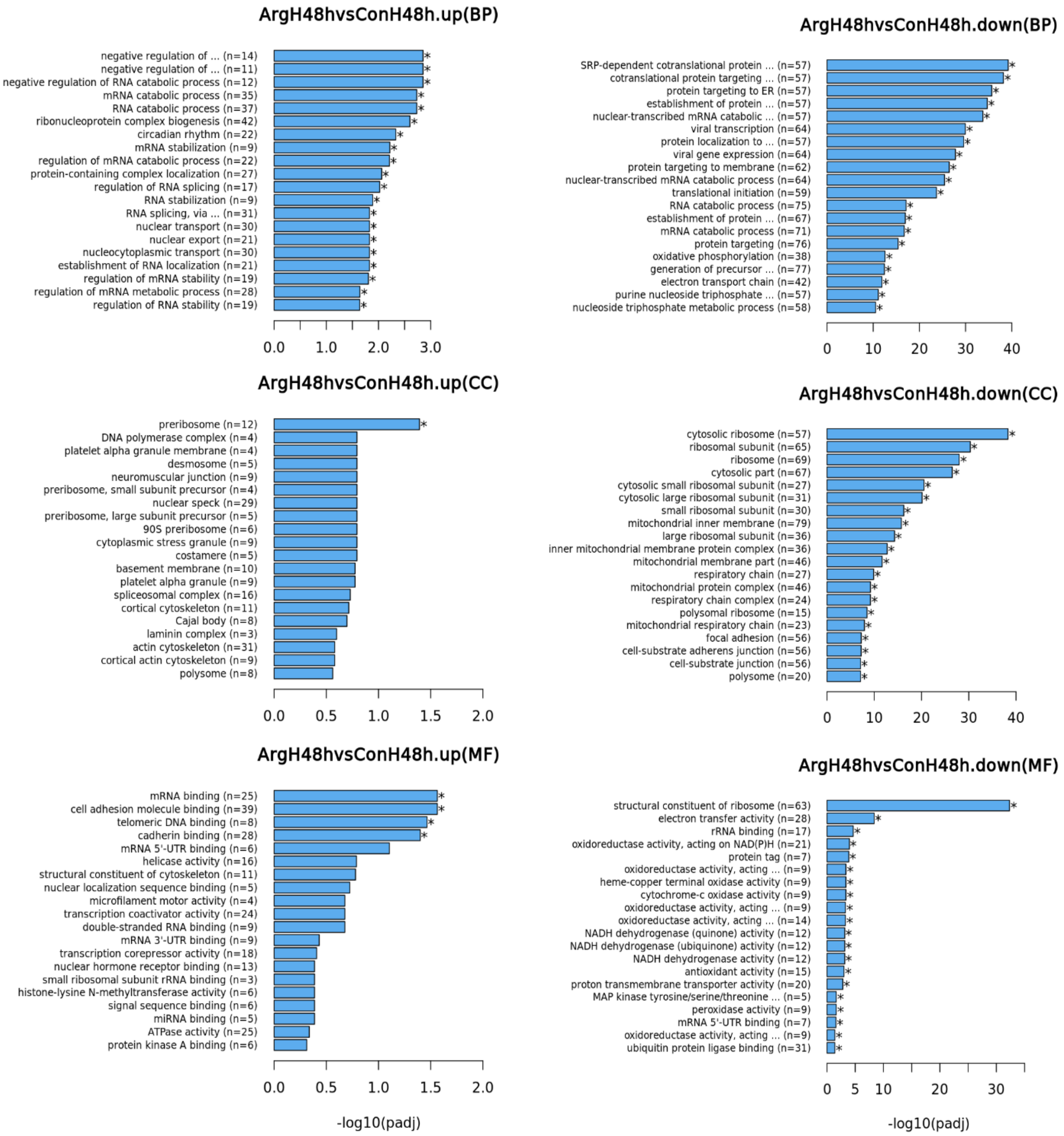

Figure S3: Top 20 upregulated and downregulated enriched gene ontology (GO) terms (biological processes/BP, molecular functions/MF, and cellular components/CC) 48 hours after Arg treatment under high glucose conditions.

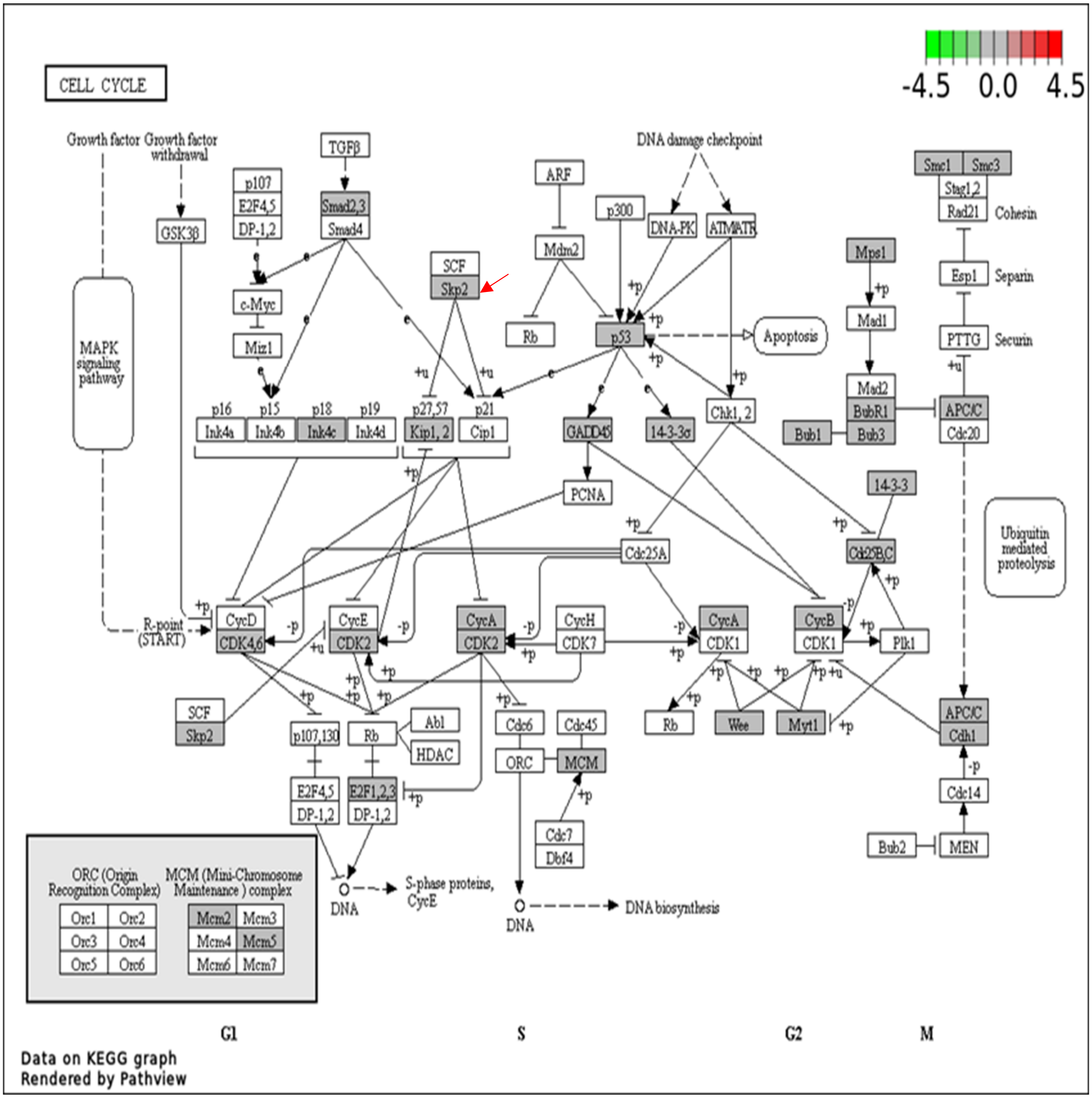

Figure S4: Significantly upregulated cell cycle pathway in Enriched Kyoto Encyclopedia of Genes and Genomes (KEGG) 24 hours after Arg treatment under high glucose conditions

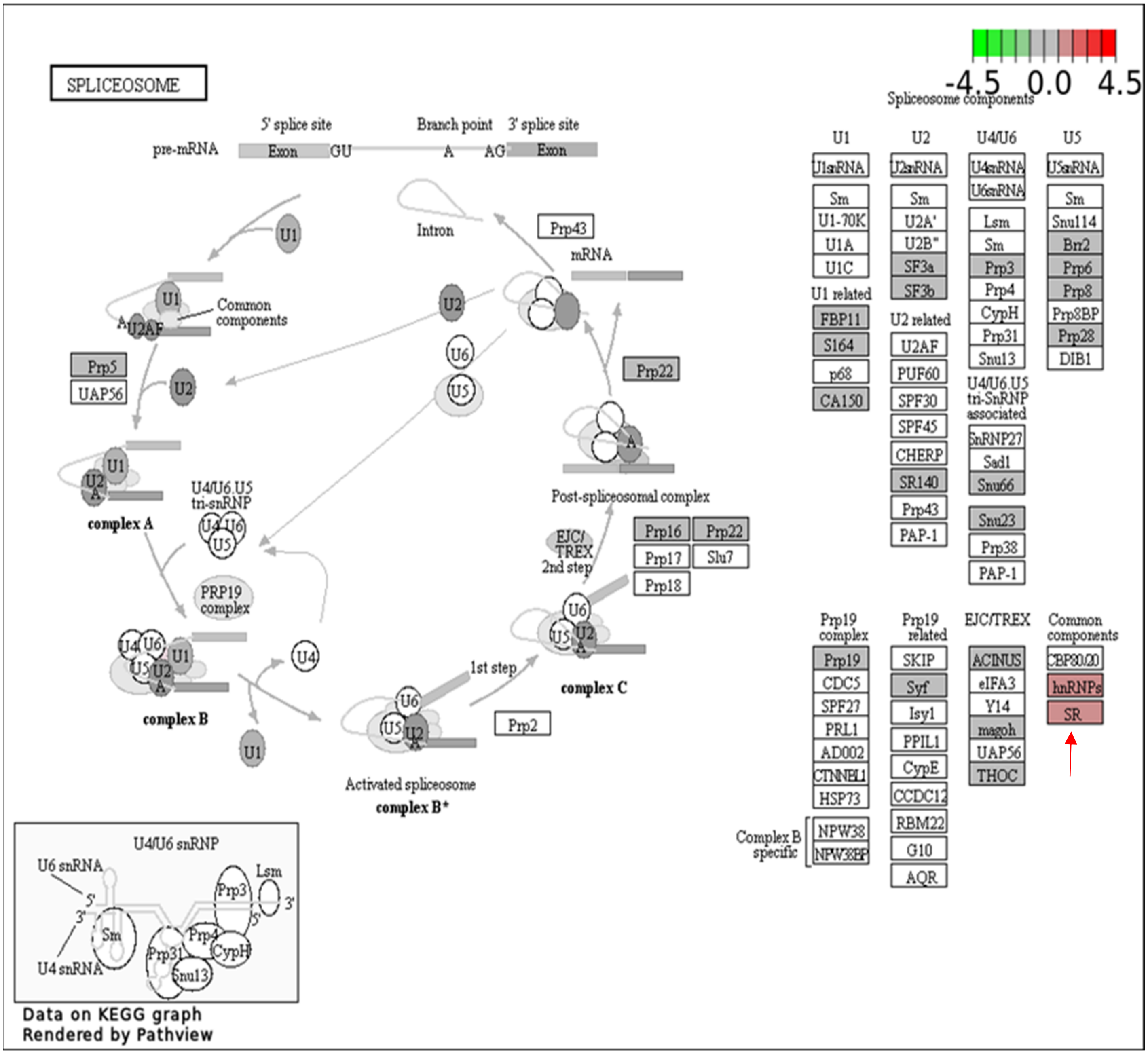

Figure S5: Significantly upregulated spliceosome pathway in Enriched Kyoto Encyclopedia of Genes and Genomes (KEGG) 24 hours after Arg treatment under high glucose conditions.

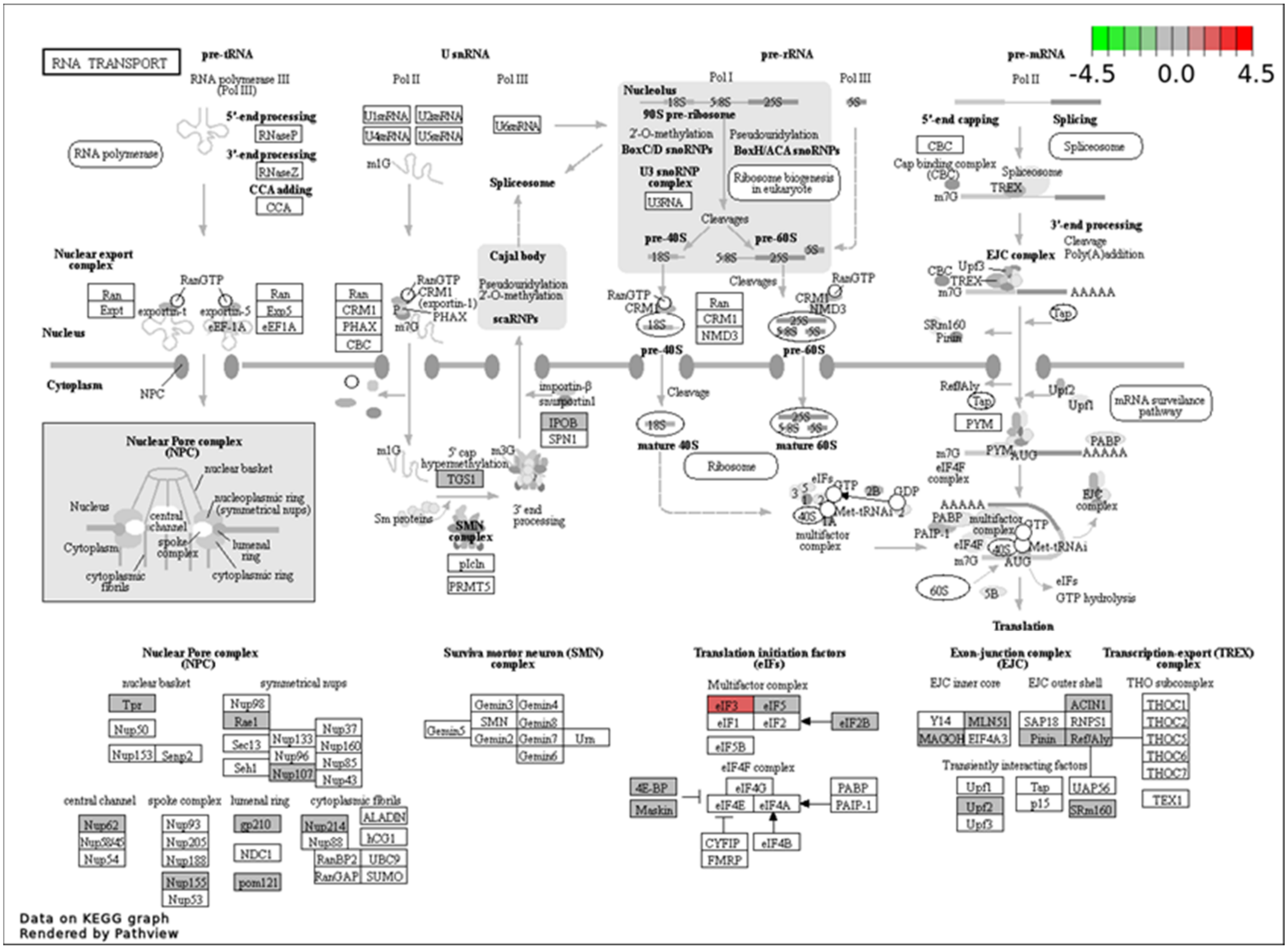

Figure S6: Significantly upregulated RNA transport in Enriched Kyoto Encyclopedia of Genes and Genomes (KEGG) pathway 24 hours after Arg treatment under high glucose conditions.

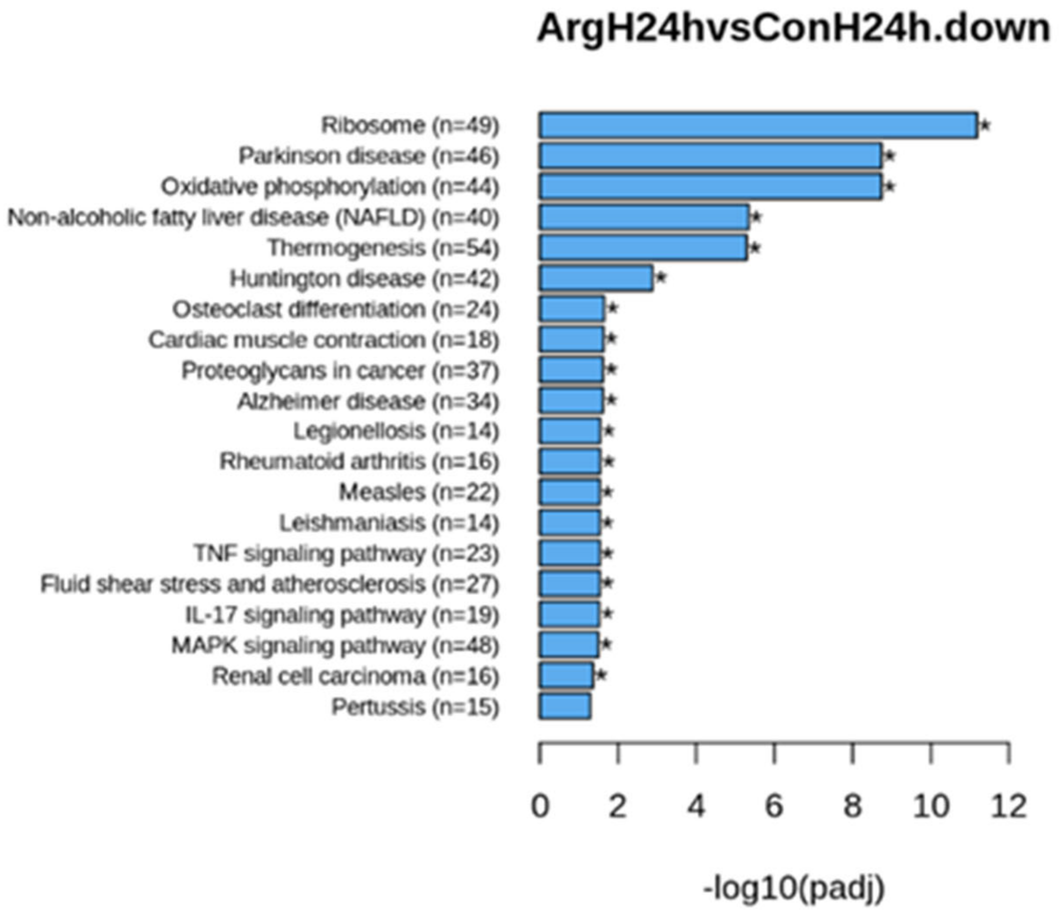

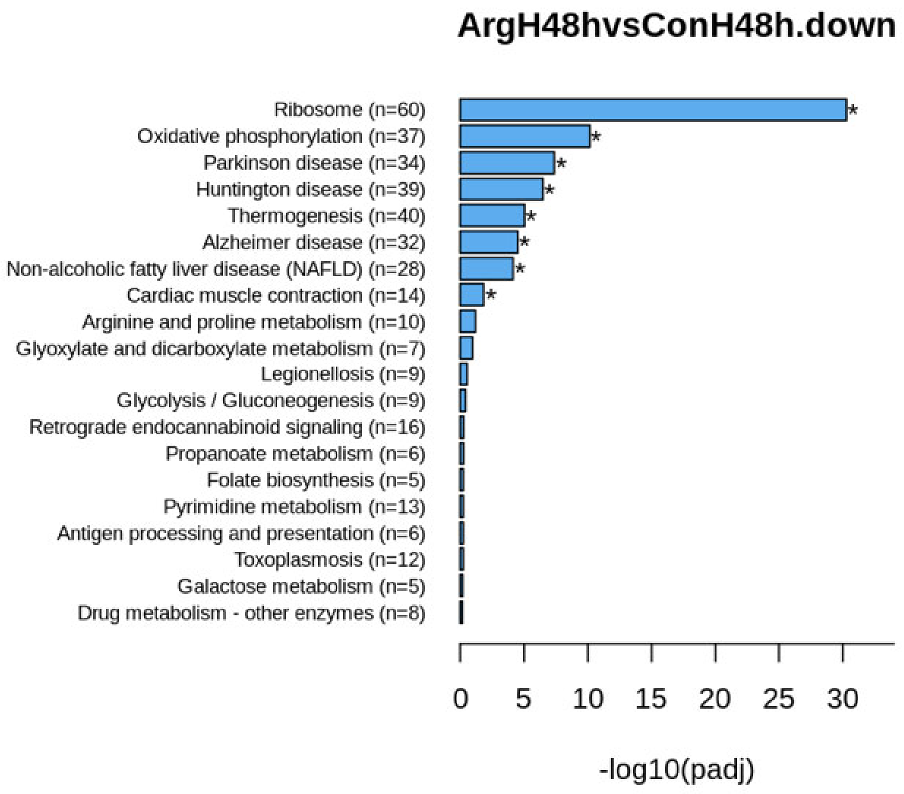

Figure S7: Top 20 downregulated enriched Kyoto Encyclopedia of Genes and Genomes (KEGG) pathways 24 and 48 hours after Arg treatment under high glucose conditions.
